# Protein-based bandpass filters for controlling cellular signaling with chemical inputs

**DOI:** 10.1038/s41589-023-01463-7

**Published:** 2023-11-13

**Authors:** Sailan Shui, Leo Scheller, Bruno E. Correia

**Affiliations:** 1 Laboratory of Protein Design and Immunoengineering (LPDI)-STI-EPFL, Lausanne, Switzerland; 2 Swiss Institute of Bioinformatics (SIB), Lausanne, Switzerland

**Keywords:** Synthetic biology, Protein design, Cell signalling, Small molecules

## Abstract

Biological signal processing is vital for cellular function. Similar to electronic circuits, cells process signals via integrated mechanisms. In electronics, bandpass filters transmit frequencies with defined ranges, but protein-based counterparts for controlled responses are lacking in engineered biological systems. Here, we rationally design protein-based, chemically responsive bandpass filters (CBPs) showing OFF-ON-OFF patterns that respond to chemical concentrations within a specific range and reject concentrations outside that range. Employing structure-based strategies, we designed a heterodimeric construct that dimerizes in response to low concentrations of a small molecule (ON), and dissociates at high concentrations of the same molecule (OFF). The CBPs have a multidomain architecture in which we used known drug receptors, a computationally designed protein binder and small-molecule inhibitors. This modular system allows fine-tuning for optimal performance in terms of bandwidth, response, cutoff and fold changes. The CBPs were used to regulate cell surface receptor signaling pathways to control cellular activities in engineered cells.

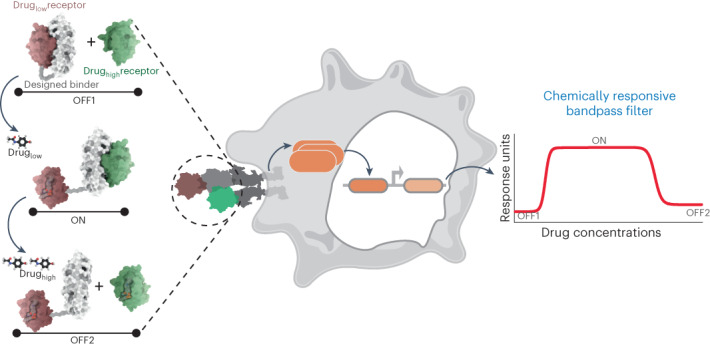

## Abstract (web summary)

Development of chemically responsive bandpass filters mimics the signal-processing abilities of electronic circuits in mammalian cells by responding to chemical concentrations within a specific range and rejecting ones outside that range.

## Main

Cells are sophisticated signal-processing units that respond and adapt to environmental and internal signals^1^. Synthetic biologists strive to engineer cells that process signals in a systematic, predictable, controllable and integrated manner^2^. Chemically responsive protein modules are particularly useful for engineering artificial cellular activities controlled by external cues^1,3^. Currently available protein switches sense a specific chemical and control the activity of biological circuits through induced protein–protein association (for example, rapamycin-induced FKBP–FRB dimerization^4^) or dissociation (chemically disruptable heterodimers^5^). Protein switches are capable of up- or downregulation of cellular activities, but require a high dilution of the chemicals to revert the controlled cellular activity from ON to OFF, or vice versa^3^. However, cells perceive and respond to temporal and spatial stimulations in a timely and efficient manner^6^. For instance, processes that result in pattern formation are a hallmark of such coordinated and complex behavior. Current engineered protein components mostly up- or downregulate outputs in a uniform manner, and therefore engineered cellular systems often fail in replicating such sophisticated signal-processing events.

Morphogen-driven pattern organization is a hallmark of coordination of cell behavior and signal detection of gradient concentrations. To engineer morphogen function, many efforts have been focused on developing band-detecting systems that mimic electronic bandpass filters^7^. An electronic bandpass filter can pass frequencies within a defined range, and reject frequencies outside that range. Bandpass filters have been engineered in biological systems by tuning the affinity of a trans-repressor for the acyl-homoserine lactone signaling molecule and dosing with different plasmid copies^8^. Bandpass filters were also designed through a dual approach of positive and negative genetic selections for β-lactamase activity. Cellular β-lactamase activity was directed by an IPTG-inducible promoter, regulating cell growth exclusively within a defined range in the presence of the β-lactam antibiotics ampicillin and tetracycline^9^. The cellular β-lactamase activity-detecting bandpass filter was used to demonstrate the effects of multiple morphogen gradients on cell survival^10^; furthermore, this bandpass filter has been adapted to select protein–protein interactions of different binding strength^11^. Artificial bacterial transcription factors have also been designed to sense and respond to d-fucose and IPTG to mimic band-detecting behavior^12^. Other biological networks with band-detecting characteristics have been developed on the basis of transcription factors, including detection of l-arabinose^13^ and nisin^14^. Similar work has also been done in mammalian systems, where a synthetic genetic network was constructed with bandpass characteristics in which output expression is only ‘ON’ across a window of low concentrations of tetracycline^15^.

**Fig. 1:**
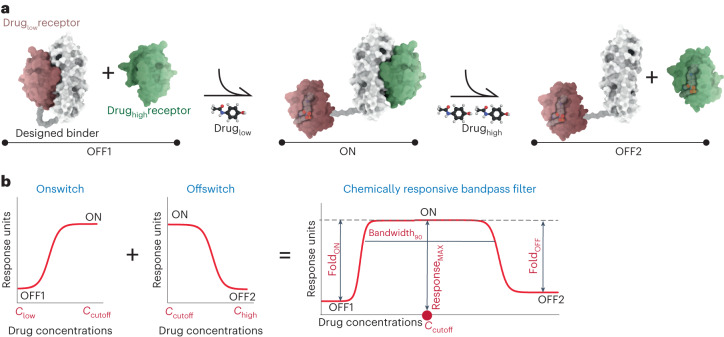
Design strategy and performance parameters of the chemically responsive protein-based bandpass filters. **a**, Schematic representation of the CBP mode of action. Each CBP contains three proteins: the designed binder (white surface representation) and two drug receptors (drug_low_ receptor in red and drug_high_ receptor in green). For low drug concentrations, CBPs switch to the ON state, and in high drug concentrations CBPs switch to the OFF2 state. **b**, Design principles of the CBPs by integrating an ON switch (from low drug concentration to cutoff drug concentration) and an OFF switch (from cutoff drug concentration to high drug concentration), and the tunable parameters of each CBP, including cutoff concentration (*C*_cutoff_, drug concentration of Response_MAX_), bandwidth (Bandwidth_90_, drug concentration range maintains ≥90% of Response_MAX_), fold changes (Fold_ON_ = Response_MAX_/Response_OFF1_, Fold_OFF_ = Response_MAX_/Response_OFF2_) and the maximum response (Response_MAX_).

Despite these examples of genetic bandpass circuitry, there are no engineered protein-based sensors that do not rely on transcriptional regulation systems that can perform such behaviors. There are also limitations in terms of the choice of transcription factors and small-molecule inducers that can be used in genetic circuits. To overcome these limitations, a generalizable approach for engineering robust protein-based bandpass filters is needed. Hence, we rationally designed CBPs that detect drug concentrations, pass concentrations within a certain range and reject concentrations beyond that range, resulting in an ‘OFF-ON-OFF’ regulatory pattern. Each CBP consists of three distinct protein components: a designed binder and a drug_low_ receptor and a drug_high_ receptor that are sensitive to drug disruption in low and high concentrations, respectively. Each CBP undergoes an ‘ON switch’ process, where the designed binder dissociates from the drug_low_ receptor at low drug concentrations and consequently binds to the drug_high_ receptor, and an ‘OFF switch’ process, where the designed binder dissociates from the drug_high_ receptor at high drug concentrations (Fig. 1a). On the basis of the unique behavior of the CBPs, we identified five key parameters, including fold changes between the ON state and two OFF states, maximum response, cutoff concentration and bandwidth, which can be tuned to sustain the activity controlled by CBP at a defined level (Fig. 1b). These protein-based CBPs are highly modular; the binding affinities between the drug receptors and the designed binder, as well as the drug sensitivities of the drug receptors, can be modulated to adjust the parameters of the CBPs. Furthermore, the designed CBPs were incorporated in cell surface receptors to regulate signaling pathways externally in a bandpass filtering fashion. Our results show the potential of rationally designed protein-based CBPs for diverse cellular applications.

## Results

### Bandpass filter design by integrating ON and OFF switches

Analogous to electronic bandpass filters, we sought to design CBPs with an OFF-ON-OFF regulatory pattern depending on the concentration of a drug. To do so, three proteins were used to construct a CBP: a designed binder that interacts with both drug receptors, a drug receptor that is dissociated from the designed binder at low drug concentrations (drug_low_ receptor) and a drug receptor that can only be dissociated at high drug concentrations (drug_high_ receptor). The designed binder is fused to the drug_low_ receptor via a (GGGGS)_3_ linker (OFF1 state, intramolecular dimerization, intermolecular dissociation), is dissociated from the drug_low_ receptor to bind to the drug_high_ receptor at low drug concentrations (ON state, intermolecular dimerization) and is dissociated from both drug receptors at high drug concentrations (OFF2 state) (Fig. 1a).

Biological activities controlled by CBPs are designed to be upregulated and then downregulated with increasing drug concentrations. We hypothesized that the up- and downregulation could be mimicked by integrating a protein-based ON switch, which turns biological activity ON from low to cutoff concentrations (from OFF1 state to ON state), and an OFF switch, which turns biological activity OFF from cutoff to high concentrations (from ON state to OFF2 state) (Fig. 1b, left). Many parameters of CBPs can be characterized and modulated. Beyond fold changes and maximum response, we also monitored the cutoff concentration indicating the highest response that can be achieved, and the bandwidth indicating the range of drug concentrations that retain the ON state (Fig. 1b, right).

### Dual-drug-responsive cell surface receptors

Bcl2 and its homologous protein, BclXL, are primarily responsible for exerting anti-apoptotic functions by binding to the BH3 domain of pro-apoptotic proteins^16–18^ (Supplementary Fig. 1a). Bcl2 and BclXL promote cell survival and are frequently upregulated in many tumors^19–21^, making them attractive targets for anticancer drug development. Over the past decades, a number of drugs have been developed. One such drug is navitoclax^22^, an experimental, orally active anticancer agent that binds both BclXL and Bcl2. In contrast, venetoclax^23^ stands out as the first-in-class (BH3 mimetic) clinically approved drug that selectively binds to Bcl2. A-1155463 (ref. ^24^) is an experimental drug that has been identified as a selective inhibitor specifically targeting BclXL (Supplementary Fig. 1b). Moreover, a computationally designed binder, LD3, presented a stabilized conformation of the BH3 domain that can bind to both BclXL and Bcl2 (ref. ^5^) (Supplementary Fig. 1c). For these reasons, we chose BclXL and Bcl2 as the design basis of the drug receptors for our CBP constructs.

**Fig. 2:**
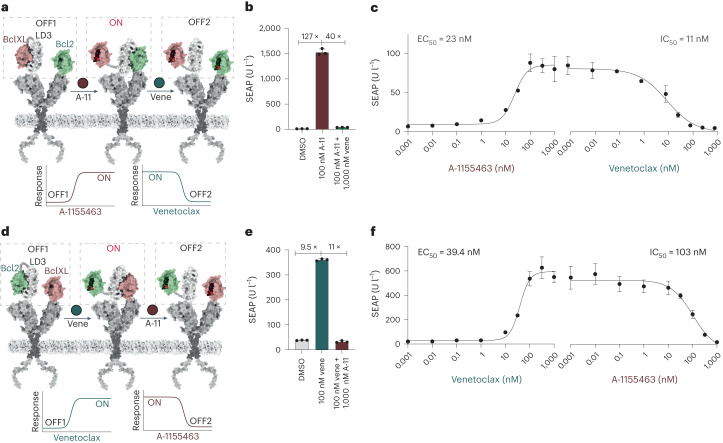
Dual-drug-controlled switches based on BclXL and Bcl2 proteins controlled by two sequential chemical inputs mimicking the CBP behavior. **a**, Scheme of Bcl2 and BclXL–LD3 complex fused to GEMS. The GEMS with the dual-drug-controlled (A-1155463, venetoclax) switch is initially in the OFF1 state and can be switched to the ON state by the BclXL inhibitor A-1155463 (A-11; red circle), and can be switched to the OFF2 state by the Bcl2 inhibitor venetoclax (vene; green circle). The curves on the bottom show the idealized output target for switching between states. **b**, Fold change of GEMS with the dual-drug-controlled (A-1155463, venetoclax) switch between no drug treatment (DMSO) versus 100 nM A-1155463 (ON) versus 100 nM A-1155463 and 1 µM venetoclax (OFF), showing SEAP expression after 24 h. Each bar represents the mean of three biological replicates ± s.d., overlaid with the original data points. **c**, Dose responses in engineered cells expressing GEMS with the dual-drug-controlled (A-1155463, venetoclax) switch. Each data point represents the mean ± s.d. of three replicates. The EC_50_ values of the ON phase and IC_50_ values of the OFF phase were calculated individually using four-parameter nonlinear regression. **d**, Scheme of BclXL and Bcl2–LD3 complex fused to GEMS. The GEMS with the dual-drug-controlled (venetoclax, A-1155463) switch can be turned to the ON state by the Bcl2 inhibitor venetoclax (vene; green circle) and OFF with the BclXL inhibitor A-1155463 (A-11; red circle). The curves on the bottom show the idealized output target for switching between states. **e**, Fold change of GEMS with the dual-drug-controlled (venetoclax, A-1155463) switch activity between no drug treatment (DMSO) versus 100 nM venetoclax (ON) versus 100 nM venetoclax and 1 µM A-1155463 (OFF), showing SEAP expression after 24 h. Each bar represents the mean of three biological replicates ± s.d., overlaid with the original data points. **f**, Dose responses in engineered cells expressing the GEMS with the dual-drug-controlled (venetoclax, A-1155463) switch. Each data point represents the mean ± s.d. of three replicates and the EC_50_ values of the ON phase and IC_50_ values of the OFF phase were calculated using four-parameter nonlinear regression. Source data

In the first step, we sought to construct a dual-drug-controlled switch to test whether the ON and OFF switching processes can be combined in our receptor architecture. We used Bcl2 and BclXL as the orthogonal drug receptors, which interact with the LD3 protein and respond to their own specific inhibitors. In such a setting, the dual-drug-controlled switch will turn ON in response to one drug and OFF in response to the other, thus mimicking bandpass filtering behavior (Supplementary Fig. 1d). The compound A-1155463 interacts with BclXL with a *K*_d_ lower than 10 pM, while its binding to Bcl2 is more than 7,000-fold weaker^24^. We confirmed that A-1155463 prevents BclXL from binding to LD3 with a half-maximum inhibitory concentration (IC_50_) of 106 nM, and does not affect the Bcl2–LD3 interaction, with a detectable IC_50_ in a surface plasmon resonance (SPR) drug competition assay (Fig. 2a). This result shows that Bcl2 is not affected by the inhibitor of BclXL. Thus, we constructed the first dual-drug-controlled switch using Bcl2 and the fused BclXL–LD3 complex, which is expected to turn ON with A-1155463 and to turn OFF with venetoclax. To test the switch behavior, we employed the generalizable extracellular molecule sensor (GEMS) platform^25^, which senses extracellular molecules and thus regulates cell surface signaling pathways (Fig. 2a). This receptor platform is based on a mutated EpoR extracellular and transmembrane domain fused to an IL-6RB intracellular domain. Dimerization of EpoR extracellular domains is controlled by N-terminally fused inducible dimerization domains, which drive IL-6RB activation and STAT3 signaling. The Bcl2 and BclXL–LD3 complexes were fused to the extracellular EpoR domains. The intramolecular interaction of BclXL–LD3 can be disrupted by A-1155463, opening up the binding site of LD3 for Bcl2 protein. The dimerization of Bcl2 and LD3 causes EpoR extracellular domain dimerization and triggers JAK/STAT3 signaling. Active STAT3 drives the expression of a reporter protein (secreted alkaline phosphatase (SEAP)) controlled by a STAT3-dependent promoter. The GEMS with the dual-drug-controlled (A-1155463, venetoclax) switch turned on reporter gene expression with a 100-fold increase in the presence of 100 nM A-1155463, and was effectively shut down by adding 1 µM venetoclax (Fig. 2b). The dual use of A-1155463 and venetoclax showed a slight effect on reporter gene production (Supplementary Fig. 2b) but not on cell proliferation (Supplementary Fig. 2c). The dose response had a half-maximum effective concentration (EC_50_) of 23 nM in the ON phase and an IC_50_ of 11 nM in the OFF phase (Fig. 2c).

We constructed the second dual-drug-controlled switch using BclXL and Bcl2–LD3 complex, which can be turned ON by venetoclax and turned OFF by A-1155463 (Fig. 2d). Venetoclax selectively binds to Bcl2 rather than to BclXL, with 500-fold stronger affinity^23^. Venetoclax also shows great selectivity in inhibiting the LD3–Bcl2 interaction (IC_50_ = 67 nM) while not affecting the BclXL–LD3 interaction (Supplementary Fig. 2d). These GEMS with dual-drug-controlled (venetoclax, A-1155463) switch showed a close to tenfold increase and decrease in the ON and OFF phases, respectively (Fig. 2e). The dose response had an EC_50_ of 39 nM in the venetoclax-controlled ON phase and an IC_50_ of 103 nM in the A-1155463-controlled OFF phase (Fig. 2f). The combined use of A-1155463 and venetoclax did not cause cellular toxicity on cell proliferation, but a small decrease in reporter protein production was observed (Supplementary Fig. 2e,f).

These results show that the dual-drug-controlled switches can regulate cell surface receptor signaling in an OFF-ON-OFF pattern in response to two drugs, serving as a proof of concept for our design strategy.

### A bandpass filter responsive to one chemical input

To test whether the OFF-ON-OFF strategy translated into a single chemical input CBP design, we tested both dual-drug-controlled switches with the Bcl2 family inhibitor navitoclax, which binds to BclXL and Bcl2 with different potencies^22^. We compared navitoclax BclXL–LD3 or Bcl2–LD3 complex disruption in the GEMS platform individually, which showed approximately 4.4-fold more potent activity to BclXL than to Bcl2, and IC_50_ values of 403 nM and 1,773 nM, respectively (Supplementary Fig. 3a,b).

**Fig. 3:**
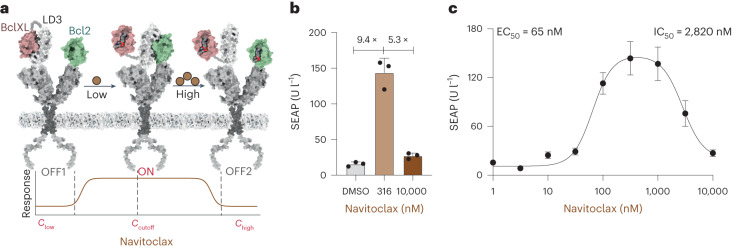
Schematic representation and characterization of CBP_navi_. **a**, Scheme of CBP_navi_ and its response to different drug concentrations. **b**, Fold change in reporter gene expression between no drug treatment (DMSO; OFF1) versus 316 nM (ON) versus high concentration 10,000 nM (OFF2), 24 h after drug addition. Each bar represents the mean of three biological replicates ± s.d., overlaid with a scatter dot plot of the original data points. **c**, Dose response to navitoclax in engineered cells expressing CBP_navi_. Each data point represents the mean ± s.d. of three biological replicates. EC_50_ of the ON phase and IC_50_ of the OFF phase were calculated using four-parameter nonlinear regression. Source data

Next, we tested a CBP responsive to navitoclax (CBP_navi_), which dissociates LD3 from the fused BclXL–LD3 complex to interact with Bcl2 at low concentrations, and dissociates LD3 from Bcl2 at high concentrations (Fig. 3a). CBP_navi_ was used to control receptor activity in the GEMS platform, which can be maximally activated at a cutoff concentration of 320 nM with an approximately ninefold increase in reporter gene expression, and can be turned OFF at 10 µM (Fig. 3b), which does not interfere with reporter gene expression or cell proliferation (Supplementary Fig. 3c,d). We also characterized the ON and OFF kinetics of CBP_navi_, showing an EC_50_ of 65 nM in the ON phase and an IC_50_ of 2,820 nM in the OFF phase (Fig. 3c).

The CBP_navi_ was constructed on the basis of the different drug sensitivities between BclXL and Bcl2 towards navitoclax, implying that rational design of drug receptors with different drug sensitivities can lead to a general strategy for the engineering of bandpass filter protein devices.

### Rational design of CBPs

**Fig. 4:**
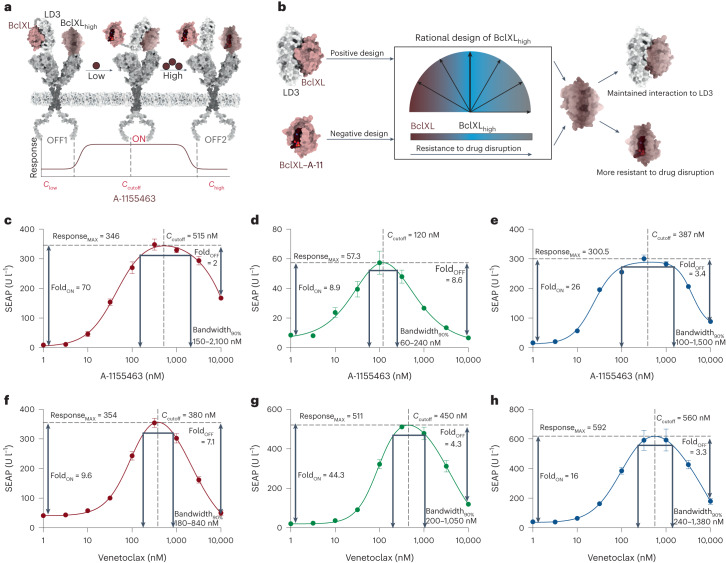
Rational design of CBPs that respond to small-molecule drugs. **a**, Scheme of CBP_A11_ and its response in different ranges of drug concentration. **b**, BclXL_high_ designs were generated to perform bandpass filtering functions in response to different concentrations of A-1155463. **c**–**e**, Dose responses of CBP_A11_-v1 (**c**, red), CBP_A11_-v2 (**d**, green) and CBP_A11_-v3 (**e**, blue) in engineered cells. Each data point represents the mean ± s.d. of three replicates and the curves were calculated using bell-shaped fitting. **f–h**, Dose responses of CBP_vene_-v1 (**f**, red), CBP_vene_-v2 (**g**, green) and CBP_vene_-v3 (**h**, blue) in engineered cells. Each data point represents the mean ± s.d. of three replicates and the curves were calculated using bell-shaped fitting. Source data

To rationally design a CBP responding to a chemical input, we used BclXL as the drug_low_ receptor, LD3 as the designed binder and a designed BclXL variant as the drug_high_ receptor (referred to as BclXL_high_) to construct a CBP responsive to BclXL inhibitors (Fig. 4a). To design BclXL_high_, we used a multistate design computational protocol described elsewhere^26^ (Methods) and generated six variants, BclXL_high_-v(1–6), which retain binding to LD3 but have higher resistance to A-1155463 (Fig. 4b and Supplementary Table 1). We screened the candidate receptors in the CBP architecture and we observed that BclXL_high_-v(4–6) maintained strong resistance to A-1155463, while BclXL_high_-v(1–3) showed a notable decrease when the drug concentration reached 1 µM (Supplementary Fig. 4a). Thus, BclXL_high_-v(1–3) were chosen as the functional drug_high_ receptors, resulting in three A-1155463-controlled CBPs (referred to as CBP_A11_-v(1–3)) (Fig. 4c–e). A-1155463 does not perturb reporter gene expression or cell survival at the highest concentration of 10 µM (Supplementary Fig. 4b,c).

All CBP_A11_-v(1–3) showed distinctive ON phases and OFF phases in response to different concentration ranges of A-1155463. CBP_A11_-v1 and CBP_A11_-v3 showed an ON phase with a Fold_ON_ of 70-fold and 26-fold reporter expression over OFF1, respectively; however, they did not efficiently shut down in the OFF2 phase (Fig. 4c,e). CBP_A11_-v2 demonstrated Fold_ON_ of ninefold with 100 nM A-1155463, but shut down to background level at 10 µM (Fig. 4d). We hypothesize that the fold changes (Fold_ON_ and Fold_OFF_) are related to the binding affinities between the drug_high_ receptor and the LD3 protein (BclXL_high_-v1: 44 nM and BclXL_high_-v3: 37 nM versus BclXL_high_-v2: 159 nM), with the stronger binder contributing to the higher Fold_ON_ and lower Fold_OFF_ (Supplementary Fig. 4d). The tighter interaction of BclXL_high_-v1–LD3 and BclXL_high_-v3–LD3 requires a higher drug concentration to dissociate the intermolecular interaction, resulting in a stronger activation and an ineffective disruption in OFF2 phases. Conversely, the weak interaction between BclXL_high_-v2 and LD3 resulted in a lower receptor activation but complete disruption in the OFF phase of CBP_A11_-v2 (Fig. 4d).

Next, we characterized the cutoff concentration (*C*_cutoff_) and bandwidth (Bandwidth_90%_) parameters of CBP_A11_-v(1–3). CBP_A11_-v2 showed the lowest *C*_cutoff_ at around 120 nM, and CBP_A11_-v1 and CBP_A11_-v3 both have a *C*_cutoff_ above 300 nM (Fig. 4c–e). CBP_A11_-v2 has the narrowest Bandwidth_90%_ of 80–240 nM. In contrast, CBP_A11_-v1 has a Bandwidth_90%_ of 150 nM to 2,100 nM (Fig. 4c–e). These data show that the drug sensitivity of BclXL_high_-v(1–3) to A-1155463 (Supplementary Fig. 4e) is well translated into the differences in the *C*_cutoff_ and Bandwidth_90%_ of CBP_A11_-v(1–3). The more sensitive BclXL_high_ compared to wild-type BclXL, the lower the *C*_cutoff_ and Response_MAX_, and the narrower the Bandwidth_90%_. For instance, BclXL_high_-v1 has the strongest drug resistance to A-1155463, which showed a correspondingly high *C*_cutoff_ and the widest Bandwidth_90%_.

Next, we constructed a venetoclax (Federal Drug Administration-approved Bcl2 inhibitor) responsive CBP (CBP_vene_) using Bcl2, LD3 and a designed drug_high_ receptor based on Bcl2 (Bcl2_high_) (Supplementary Fig. 5a). Using multistate design, we generated eight Bcl2_high_ variants (referred to as Bcl2_high_-v(1–8)), which were designed for lower affinity to venetoclax compared to native Bcl2 (Supplementary Fig. 5b and Supplementary Table 2). CBP constructs including Bcl2_high_-v(1–8) were screened, and Bcl2_high_-v2, Bcl2_high_-v4 and Bcl2_high_-v5 presented the desired behavior, with a sharp decrease when venetoclax exceeds 100 nM (Supplementary Fig. 5c). We confirmed that 10 µM venetoclax is not toxic for reporter gene expression and cell proliferation (Supplementary Fig. 5d,e).

CBP_vene_-v2 showed a Fold_ON_ and a Fold_OFF_ of about ninefold (Fig. 4f). CBP_vene_-v4 has a higher Fold_ON_ (44-fold) but does not completely shut off at 10 µM, with a Fold_OFF_ of 3.4 (Fig. 4g). CBP_vene_-v5 displayed a Fold_ON_ of 16-fold, the highest *C*_cutoff_ at 560 nM and the widest Bandwidth_90%_, with a range of 240–1,380 nM (Fig. 4h). Notably, the binding affinities between Bcl2_high_-v(2,4,5) and LD3 are close (Supplementary Fig. 6a), indicating that their drug resistance is critical for regulating the *C*_cutoff_ and Bandwidth_90%_. For instance, the Bcl2_high_-v4 is more resistant than Bcl2_high_-v2 (Supplementary Fig. 6b), leading to the higher *C*_cutoff_ (CBP_vene_-v4: 450 nM versus CBP_vene_-v2: 380 nM) and the wider Bandwidth_90%_ (CBP_vene_-v4: 200–1,050 nM versus CBP_vene_-v2: 180–840 nM).

We developed CBPs controlled by BclXL and Bcl2 inhibitors by rationally designing drug_high_ receptors with reduced drug sensitivities and using a multidomain architecture that can provide output analogous to bandpass filters.

### Tuning CBPs towards lower drug demand and larger bandwidth

We observed that CBP_A11_-v1 and CBP_A11_-v3, or CBP_vene_-v4 and CBP_vene_-v5, cannot be fully turned off at the highest drug concentration of 10 µM. However, very high drug concentrations may cause unwanted side effects due to toxicity. To improve the performance of these CBPs, we tuned the binding affinities between LD3 and the two drug receptors to lower their *C*_cutoff_ and increase the shut-down efficiency.

**Fig. 5:**
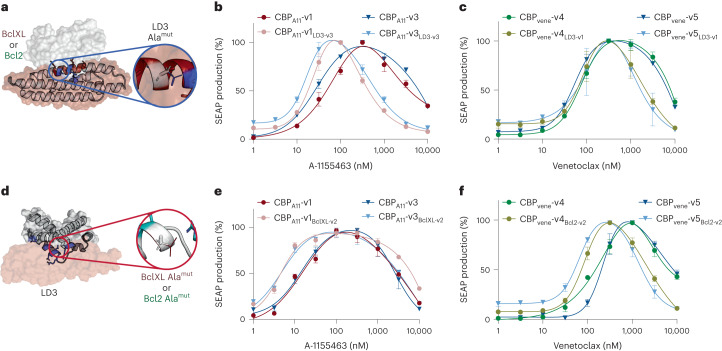
Rational tuning of binding affinities between the LD3 binder and drug receptors can further modulate the characteristics of CBPs. **a**, Rational tuning of binding affinities between LD3 (in red) and the drug receptor (BclXL or Bcl2 in white surface). Positions were selected and mutated to alanine (Ala^mut^) on LD3 to decrease the binding affinity. **b**, Normalized dose responses of CBP_A11_-v1 and CBP_A11_-v3 with and without LD3-v3. **c**, Normalized dose responses of CBP_vene_-v4 and CBP_vene_-v5 with and without LD3-v1. **d**, Scheme of the rational tuning of binding affinities between LD3 and the drug receptor by alanine scanning on BclXL or Bcl2 proteins. **e**, Normalized drug dose-dependent responses of CBP_A11_-v1 (red) and CBP_A11_-v3 (blue) compared with CBP_A11_-v1_BclXL-v2_ (pink) and CBP_A11_-v3_BclXL-v2_ (turquoise) in engineered cells. **f**, Normalized drug dose-dependent responses of CBP_vene_-v4 (green) and CBP_vene_-v5 (blue) compared with CBP_vene_-v4_Bcl2-v2_ (asparagus) and CBP_vene_-v5_Bcl2-v2_ (turquoise) in engineered cells. In **b**, **c**, **e** and **f** each data point represents the mean ± s.d. of three replicates and the curves were computed using bell-shaped fitting. Source data

Previously, we showed that mutations in the binding region of LD3 weaken the interaction of BclXL–LD3, thus lowering the drug demand to dissociate BclXL–LD3 (ref. ^26^) (Fig. 5a). We expect to obtain the same effect of such mutations in the context of the CBPs, to lower drug concentrations to turn ON and shut OFF the CBPs. We replaced LD3 in CBP_A11_-v(1–3) with LD3-v(1–3) to test whether they respond to lower concentrations of A-1155463. LD3-v3-based designs successfully shifted the *C*_cutoff_ of all three CBP_A11_-v(1–3) to lower drug concentrations (Supplementary Table 3 and Supplementary Fig. 7a–c). CBP_A11_-v1_LD3-v3_ and CBP_A11_-v3_LD3-v3_ were the best performers in terms of fold change, and both shifted their *C*_cutoff_ from above 500 nM to below 100 nM, and effectively shut down at 10 µM (Fig. 5b). However, the new LD3-v3-based CBPs also showed a lower level of ON response (Supplementary Fig. 7d,e).

We also replaced LD3 with LD3-v(1–3) in CBP_vene_-v(2,4,5) to test whether they respond to lower concentration of venetoclax. Unlike CBP_A11_-v(1–3), LD3-v3 replacement in CBP_vene_-v(2,4,5) showed leaky background activities, which failed to maintain the bandpass filtering behavior (Supplementary Fig. 8a–c). The LD3-v1 replacement shifted both CBP_vene_-v4_LD3-v1_ and CBP_vene_-v5_LD3-v1_ to lower drug concentration ranges, with no notable decrease in *C*_cutoff_ values compared to CBP_vene_-v4 or CBP_vene_-v5, respectively (Fig. 5c and Supplementary Fig. 8d,e). We found that the binding affinity of the LD3-v3–BclXL complex in CBP_A11_-v1_LD3-v3_ and CBP_A11_-v3_LD3-v3_ is 5,000-fold weaker compared to the wild-type LD3–BclXL complex^26^, which can drastically lower the *C*_cutoff_ of CBP_A11_-v1 and CBP_A11_-v3. However, the binding affinity of LD3-v1–Bcl2 in CBP_vene_-v4_LD3-v1_ and CBP_vene_-v5_LD3-v1_ is less than 200-fold weaker than LD3–Bcl2 (ref. ^26^), which was not capable of shifting the *C*_cutoff_ values substantially. We confirmed that the tuning of LD3 led to adjustment of the response of CBP_A11_ and CBP_vene_ to lower drug concentrations.

Next, we tested whether we could engineer the drug_low_ receptor to increase the sensitivity of the OFF1 to ON regulatory phase (Fig. 5d). Since the switch from ON to OFF2 should not be affected, we hypothesized that this strategy could also increase bandwidth. We scanned residues at the interface of the BclXL–LD3 complex, selected three residues on BclXL and mutated them individually to alanine (Methods and Supplementary Table 4). Among the BclXL variants (referred to as BclXL-v(1–3)), BclXL-v2 emerged as the best candidate, since BclXL-v1 and BclXL-v3 failed to function as bandpass filters in a CBP_A11_ setting (Supplementary Fig. 9a–c). We found that both CBP_A11_-v1_BclXL-v2_ and CBP_A11_-v3_BclXL-v2_ showed similar *C*_cutoff_ values and a wider bandwidth compared to CBP_A11_-v1 and CBP_A11_-v3, respectively (Fig. 5e). While OFF-phase kinetics remained similar, CBP_A11_-v1_BclXL-v2_ and CBP_A11_-v3_BclXL-v2_ turned ON at lower drug concentrations than CBP_A11_-v1 and CBP_A11_-v3, reflecting the weaker interaction of BclXL-v2–LD3 than BclXL–LD3 (Supplementary Fig. 9d,e).

Applying the same principle to CBP_vene_-v(2,4,5), we introduced alanine mutations into Bcl2 (Methods and Supplementary Table 5), referred to as Bcl2-v(1–6). On the basis of the screening results (Supplementary Fig. 10a–c), Bcl2-v2 was used for constructing CBP_vene_-v4_Bcl2-v2_ and CBP_vene_-v5_Bcl2-v2_, which demonstrated a lower *C*_cutoff_ and unexpectedly also shut down in lower concentrations compared to CBP_vene_-v4 and CBP_vene_-v5, respectively (Fig. 5f and Supplementary Fig. 10d,e).

Altogether, we showed improved performance of CBP_A11_ and CBP_vene_, with better Fold_OFF_ values and lower *C*_cutoff_ values, by rationally tuning the binding affinities between LD3 and the two drug receptors. We also redesigned the drug_low_ receptors of CBP_A11_ and CBP_vene_, BclXL and Bcl2, respectively, which shifted the ON regulatory phase to lower drug concentration ranges to tune the bandwidth and *C*_cutoff_ values of CBPs.

## Discussion

In contrast to electrical devices, cells as biological computing units use chemical inputs and biological molecules for signal processing. Protein switches have been designed to turn cellular activity ON or OFF; however, protein modules that perform bandpass filter behavior independent of engineered transcription factors remain unexplored. Here, we use computational protein design to develop protein-based bandpass filters with tunable behavior that detect drug concentrations and regulate cellular activities in an OFF-ON-OFF pattern.

First, we mimicked the behavior of bandpass filters by combining two drugs as ON and OFF signals. This approach has been attempted using engineered bacterial transcription factors to respond to two chemicals^12^; however, it lacks transferability when different transcription factors or controlled genetic circuits are required. We presented a new strategy to engineer protein-based bandpass filters by rationally designing a drug_high_ receptor that remains bound to the designed binder (ON) and can only be dissociated at high drug concentrations (OFF). In this way, each CBP responds to a single chemical input and senses the ON and OFF signals dependent on the chemical concentrations. In total, we designed ten CBPs based on BclXL and Bcl2 inhibitors producing different cutoff concentrations, from below 100 nM (for example, CBP_A11_-v1_LD3-v3_) to above 500 nM (for example, CBP_vene_-v5), as well as regulatory bandwidths with different ranges (for example, 60–240 nM for CBP_A11_-v2 versus 100–1,500 nM for CBP_A11_-v3). By rationally mutating the designed binder (LD3), CBPs can be tuned to perform with higher drug sensitivity in the trade-off with maximal response. We also demonstrated that by modulating different drug sensitivities of drug_low_ receptors, the greater the difference in drug sensitivity between drug_high_ receptor and drug_low_ receptor, the larger the bandwidth. These results demonstrate that rational protein design can be used to design and tune CBPs.

Protein-based CBPs function by induced protein–protein interactions, which can be modular and less restricted than intracellular genetic circuit-based bandpass filters^27^. While previous synthetic bandpass filter systems sensed small molecules and produced a transcriptional response^8,12,15^, they did not enable the control of other cell functions, such as signaling pathways. We utilized the designed CBPs to control engineered cytokine receptors that detect extracellular signals and regulate intracellular JAK/STAT3 signaling pathways. This application demonstrated the potential of CBPs in receptor engineering for sensing external cues, which is a first step towards sensing peptide or protein targets with bandpass behavior. Such systems could ultimately lead to the integration of bandpass filters into engineered cell communication networks for synthetic morphology or other autonomously controlled cell consortia^28,29^.

CBP-regulated systems have defined ranges of drug concentrations to sustain specific activity levels, in contrast to the simpler ON/OFF protein switch-based systems. For instance, the CBP_A11_-v1 can sustain 90% of receptor activation within drug concentrations from 150 nM to 2.1 µM. As a possible safety feature, exceeding the cutoff concentration could serve as a negative feedback to tune down the output in potential therapeutic applications. This drug concentration-dependent pattern of CBPs can be applied in controlling biological or therapeutic activities that require self-regulatory feedback from their inputs. We envision that the CBPs will be valuable components to control signal processing and to engineer sophisticated cellular functions.

## Methods

### Multistate design of single-drug reversible BclXL and Bcl2

As reported previously, a set of residues in the receptor protein (BclXL/Bcl2) binding site was selected for redesign^26^. From this set, a number of mutations were evaluated for binding energy to the binder protein (LD3) (positive design) or the drug (negative design). Afterwards, all mutations were ranked according to the difference in energy between the positive design and the negative design. The structure of BclXL bound to A-1155463 (PDB 4QVX) was used for the negative design strategy, while the model of BclXL bound to LD3 (based on the BclXL–BIM BH3 structure with PDB 3FDL) was used for positive design. Six BclXL residues in the binding site of A-1155463 (E98, R102, F105, T109, S145 and A149) were manually selected for redesign due to their proximity to drug moieties and relative distance to LD3 in the positive design structure. Each of these residues was allowed to mutate to residues with similar size/properties, restricted to a maximum of two simultaneous mutations from wild type: E98: {E/S}, R102: {F/R/K/D/E/H}, F105: {F/L/V/I/A}; T109: {S/A/T/L/V}; S145: {S/D/E/V/A}; A149: {V/A/L/I}. Six sequences, BclXL_high_-v1 (T109L, A149L), BclXL_high_-v2 (A149V), BclXL_high_-v3 (R102F, T109V), BclXL_high_-v4 (E98S,R102E, F105I), BclXL_high_-v5 (R102E, F105I, T109L) and BclXL_high_-v6 (E98S, R102E, F105I, T109L), were generated to test for single-drug reversible BclXL. All sequences are listed in Supplementary Table 1.

The structure of Bcl2 bound to venetoclax (PDB 6O0K) was used for the negative design strategy, while the model of Bcl2 bound to LD3 (PDB 6IWB) was used for positive design. Five Bcl2 residues in the binding site of venetoclax (A100, D103, V148, V156 and Y202) were manually selected for redesign due to their closeness to drug moieties and relative distance to LD3 in the positive design structure. Each of these residues was allowed to mutate to amino acids with similar size/properties, restricted to a maximum of two simultaneous mutations from wild type: A100: {A/S/T/V}, D103: {D/N/E/Q/S}, V148: {V/I/L/M/T}, V156: {V/I/L/M/T} and Y202: {Y/W/F/H/R/K/Q/E}. Three sequences, srBcl2-v1(V156I, Y202H), srBcl2-v2(D103N, Y202H) and srBcl2-v3(A100T, D103S), were selected from the top results. Five sequences were generated based on the observation of venetoclax resistance in clinical trials where G101V occurs alone or together with D103Y and D103E. Hence, we constructed srBcl2-v4(G101V), srBcl2-v5(D103Y), srBcl2-v6(G101V,D103Y), srBcl2-v7(D103E) and srBcl2-v8(G101V,D103E). All sequences are listed in Supplementary Table 2.

### AlaScan on LD3 and drug receptor proteins

LD3-v(1–3) variants were designed previously^26^ and sequences of LD3-v(1–3) are listed in Supplementary Table 3. The BclXL protein in complex with LD3 was computationally redesigned for a range of decreasing binding affinities. Rosetta’s alanine scanning filter was used to evaluate the change in ΔΔ*G* for the LD3–BclXL complex (PDB 3FDL) upon mutating each of the 22 residues in the interface of BclXL to alanine. The resulting list was then sorted by the change in ΔΔ*G*, and three residues with positive levels of change in ΔΔ*G* were selected: Q90 (0.33 REU), L90 (1.17 REU) and R99 (4.78 REU), where higher Rosetta energy unit (REU) values are predicted to result in greater affinity losses. The three mutations were selected to provide a ‘gradient’ of affinities between LD3 and BclXL; sequences of BclXL-v(1–3) are listed in Supplementary Table 4. By the same principle, Rosetta’s alanine scanning filter was used to evaluate the change in ΔΔ*G* for the LD3–Bcl2 complex (PDB 6IWB) upon mutating each of the 32 residues in the interface of BclXL to alanine. The resulting list was then sorted by the change in ΔΔ*G*, and six residues with positive levels of change in ΔΔ*G* were selected: F63 (6.29 REU), V92 (1.29 REU), E95 (1.50 REU), L96 (0.88 REU), R105 (1.52 REU) and E111 (0.59 REU), where higher REU values are predicted to result in greater affinity losses. The six mutations were selected to provide a ‘gradient’ of affinities between LD3 and Bcl2; sequences of Bcl2-v(1–6) are listed in Supplementary Table 5.

### SPR assay for protein–protein binding affinities

SPR measurements were performed on a Biacore 8K device (GE Healthcare). The mutual designed binder (LD3) was immobilized on a CM5 chip (GE Life Science) as a ligand with the concentration at 5 μg ml^−1^ for 120 s contact time in pH 4.5 sodium acetate solutions. Serial dilutions of the analytes (BclXL or Bcl2 and their variants) in HBS-EP buffer (10 mM HEPES, 150 mM NaCl, 3 mM EDTA and 0.005% surfactant P20; GE Life Science) were flowed over the immobilized chips. After each injection cycle, surface regeneration was performed using 10 mM NaOH (pH 11.95). Affinities (*K*_d_) were obtained using a steady binding model of the equilibrium model with Biacore 8K evaluation software.

### SPR drug competition assay

Drug IC_50_ values for disrupting heterodimers were measured on a Biacore 8K device. A 5 µM portion of analyte was mixed with 10 µM of BclXL or Bcl2 inhibitors according to the analyte. The mixtures of analyte and drug were injected onto the LD3 immobilized channel.

### Compounds

Venetoclax (>99.9%, Chemietek, catalog no. CT-A199), A-1155463 (99.5%, Chemietek, catalog no. CT-A115) and NVP-CGM097 (100% optically pure, Chemietek, catalog no. CT-CGM097) were used directly without further purification. Venetoclax, A-1155463 and NVP-CGM097 were each dissolved in DMSO as 10 mM stocks. Stocks were aliquoted and stored at −20 °C until use.

### Cell transfection and drug treatment

HEK293T cells were maintained in DMEM medium (Thermo Fisher) with 10% FBS (Gibco) and pen/strep (Thermo Fisher) at 37 °C and 5% CO_2_ in a humidified incubator. Cells were maintained and split every 2 d at around 80% confluence.

HEK293T cells were seeded in a 96-well plate 24 h before transfection. The transfection mix in each well consisted of 3 ng of constitutive human CMV promoter driven mammalian STAT3 expression vector (P_hCMV_-STAT3-pA), 30 ng of STAT3-induced reporter expression (O_Stat3_-P_hCMVmin_-SEAP-pA) and 50 ng of expression vectors for each GEMS receptor chain (PSV40-IgK-(drug_high_ receptor)-EpoRm-IL-6RBm-pA and PSV40-IgK-(drug_low_ receptor-GGGGSX3-LD3)-EpoRm-IL-6RBm-pA). For the specific drug_high_ receptor and drug_low_ receptor used in the different cellular assays, they are indicated as wild type or with mutations. Then, plasmid DNA was mixed with 50 μl opti-MEM (Thermo Fisher) and 600 ng of polyethyleneimine (Polysciences, catalog no. 24765-1). For drug treatment experiments, drugs were added 12 h post-transfection and incubated with cells for 24 h before the SEAP reporter detection assay.

### Reporter detection assay

SEAP activity (U l^−1^) in cell culture supernatants was quantified by kinetic measurements at 405 nm (1 minute per measurement for 30 cycles) of absorbance increase due to phosphatase-mediated hydrolysis of *para*-nitrophenyl phosphate (pNPP). A 4–80 µl portion of supernatant was adjusted with water to a final volume of 80 µl, heat inactivated (30 min at 65 °C) and mixed in a 96-well plate with 100 μl of 2× SEAP buffer (20 mM homoarginine (FluorochemChemie), 1 mM MgCl_2_, 21% (v/v) diethanolamine (Sigma Aldrich, catalog no. D8885), pH 9.8) and 20 μl of substrate solution containing 20 mM pNPP (Sigma Aldrich, catalog no. 71768).

### Statistics

Binding affinities of SPR drug competition assays were calculated using three-parameter nonlinear regression in GraphPad Prism (v.8.3.0). Representative data of cell assays are presented as individual values and mean values (bars). *n* = 3 refers to biological replicates. All IC_50_ or EC_50_ values of cell assays reported were calculated using four-parameter nonlinear regression ± s.d. Bandpass features of cutoff concentrations and Bandwidth_90%_ were estimated based on the curve fitted using bell-shaped curve nonlinear regression in GraphPad Prism (v.8.3.0).

### Reporting summary

Further information on research design is available in the Nature Portfolio Reporting Summary linked to this article.

## Online content

Any methods, additional references, Nature Portfolio reporting summaries, source data, extended data, supplementary information, acknowledgements, peer review information; details of author contributions and competing interests; and statements of data and code availability are available at 10.1038/s41589-023-01463-7.

### Supplementary information

Supplementary Information Supplementary Figs. 1–10 and Tables 1–5.

Reporting Summary

Supplementary Data Statistical source data for Supplementary Figs. 2–10.

### Source data

Source Data Figs. 2–5 Statistical source data.

## Data Availability

The data supporting the findings of this study are available within the article and its Supplementary Information. Other data and reagents are available from the corresponding authors upon reasonable request. Source data are provided with this paper.
