# Supplementary material for: Protein-based bandpass filters for controlling cellular signaling with chemical inputs
Source: Nat Chem Biol. 2023 Nov 13;20(5):586–93. doi: 10.1038/s41589-023-01463-7 (PMC11062894; doi:10.1038/s41589-023-01463-7)
Supplement: Supplementary file 1 — Supplementary Figs. 1–10 and Tables 1–5. [file 41589_2023_1463_MOESM1_ESM.pdf]

nature chemical biology

Article https://doi.org/10.1038/s41589-023-01463-7

Protein-based bandpass filters for controlling cellular signaling with chemical inputs

In the format provided by the authors and unedited

Supplementary Figures

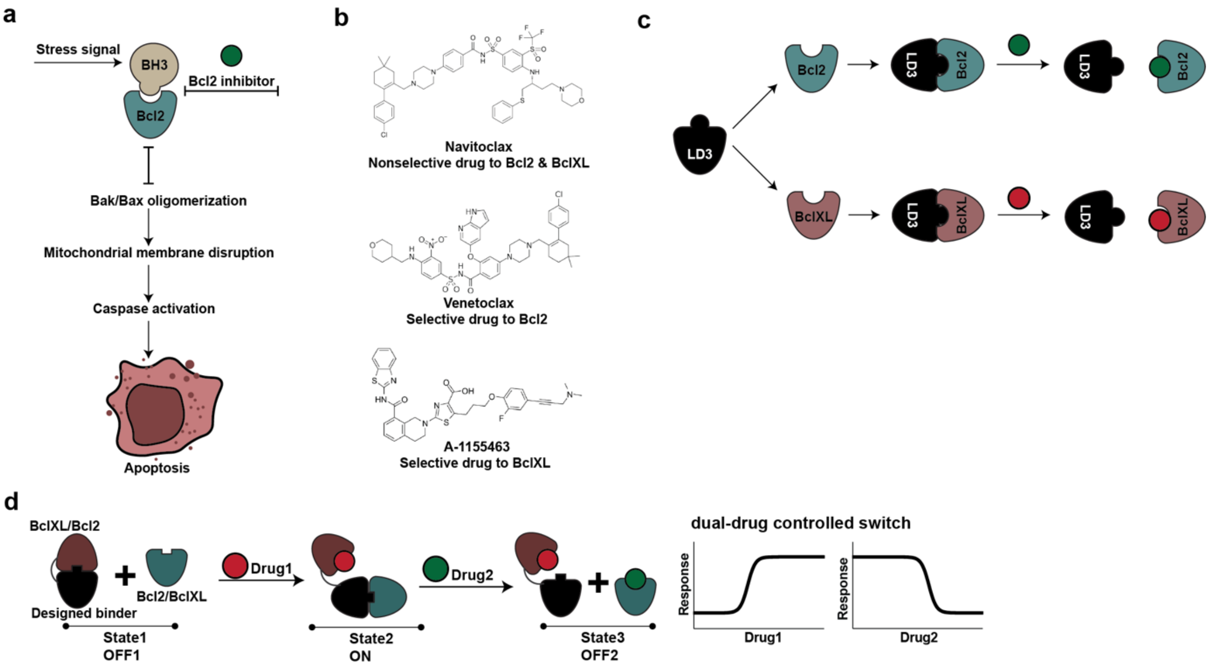

Supplementary Figure 1: BclXL and Bcl2 were selected as the design basis of drug-receptors for CBPs. a) Overview of the biological function of Bcl2/BclXL in their native context. In response to stress signals such as DNA damage, BH3-only proteins will be activated and promote apoptosis. Bcl2 and related anti-apoptotic proteins can bind to BH3 proteins and block their function. Bcl2/BclXL inhibitors (especially BH3 mimetics) disrupts the interaction between Bcl2 and BH3-only proteins to cause cell apoptosis to proceed. B) Navitoclax, Venetoclax and A-1155463 are used in this study which are known to bind Bcl2 and BclXL proteins. c) The rationally designed binder LD3 was grafted and stabilized with a BH3 motif and can interact with Bcl2 and BclXL proteins. This protein complex can be dissociated by adding specific drugs (e.g. Venetoclax for Bcl2, A-1155463 for BclXL respectively). d) A dual-drug controlled switches using Bcl2 and BclXL as orthogonal drug-receptors, which can both interact with LD3, and can be disrupted by their respective specific drugs.

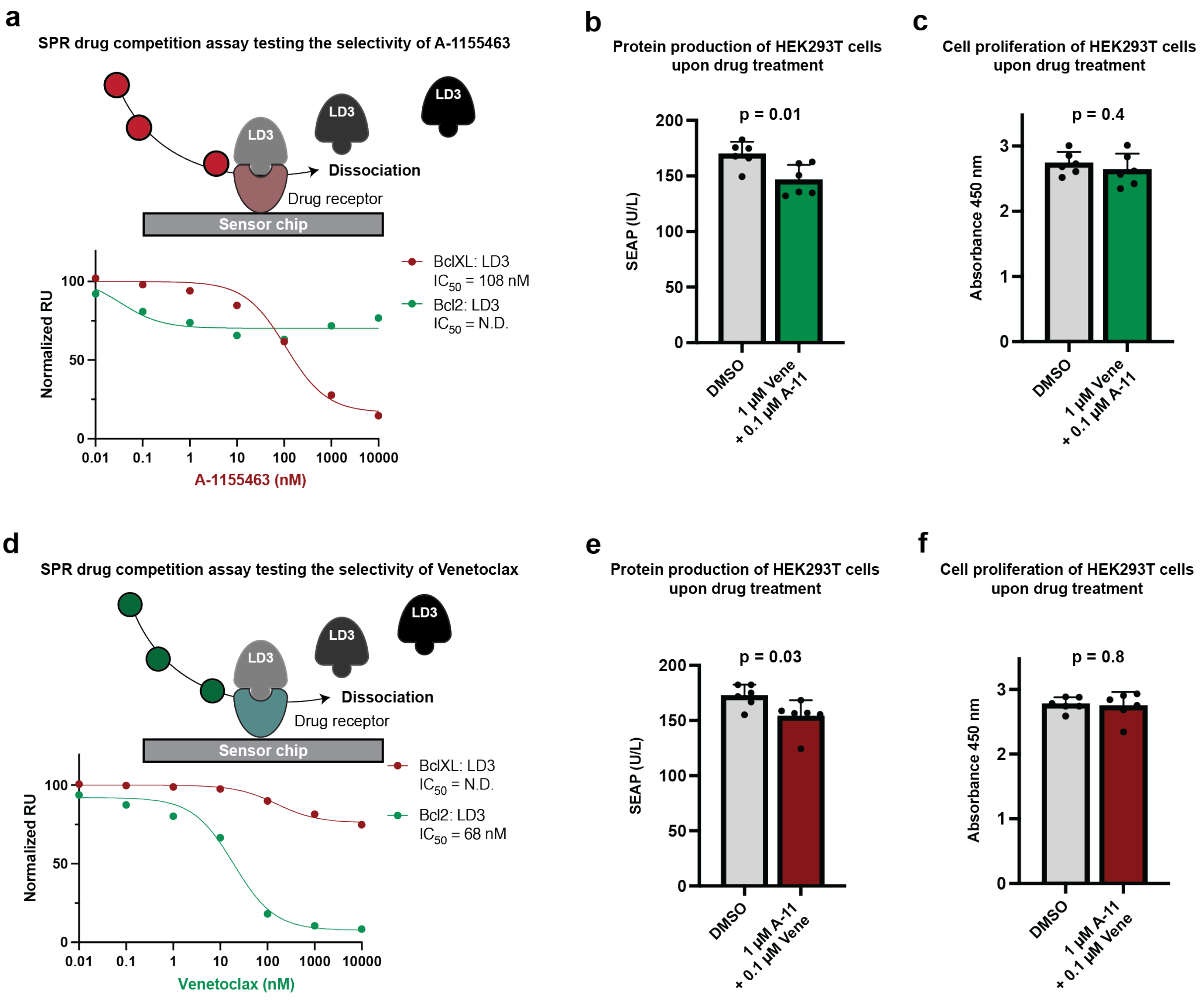

Supplementary Figure 2: Selective drugs of Bcl2 and BclXL can be used for constructing dual-drug controlled switches.

a) Apparent IC_{50}s for A-1155463 for dissociation of BclXL:LD3 and Bcl2:LD3 determined by an SPR drug competition assay. This assay is designed to test if the drug of interest can specifically disrupt the interaction between LD3 and BclXL/Bcl2. The drug-receptors (Bcl2, BclXL) are immobilized on the different channels of the sensor chip, then the binding sites are saturated with LD3. While the flux of A-1155463 (red) flows through the sensor chip, the loss of signal is used to monitor the dissociation of the protein complex. **b)** Drug effects on reporter protein expression of HEK293T cells. HEK293T cells were transfected with a plasmid for constitutive SEAP production and incubated with indicated drugs for 24 hours. SEAP quantifications were performed to measure the protein production and WST-8 was used to test the cell proliferation. Each bar represents the mean of six biological replicates ± s.d., overlaid with a scatter dot plot of the original data points. SEAP production was significantly reduced in response to the combinatorial use of A-1155463 and Venetoclax, but the effect was very small and should not affect the analysis of bandpass behavior. **c**) Drug effects on cell proliferation of HEK293T cells. WST-8 was added after 24 hours of drug incubation, and measured for absorbance at 450 nm 4 hours later. No effect on cell proliferation upon indicated drug treatment was observed. **d)** Repeat of experiment (a) for Venetoclax for dissociation of BclXL:LD3 and Bcl2:LD3. **e**) Repeat of experiment (b) with indicated drug treatment. SEAP production was significantly reduced in response to the combinatorial use of A-1155463 and Venetoclax, but the effect was very small and should not affect the analysis of bandpass behavior. **f**) Repeat of experiment (c) with indicated drug treatment. Each bar represents the mean of six biological replicates ± s.d., overlaid with a scatter dot plot of the original data points. Unpaired t-tests were used for statistical analysis.

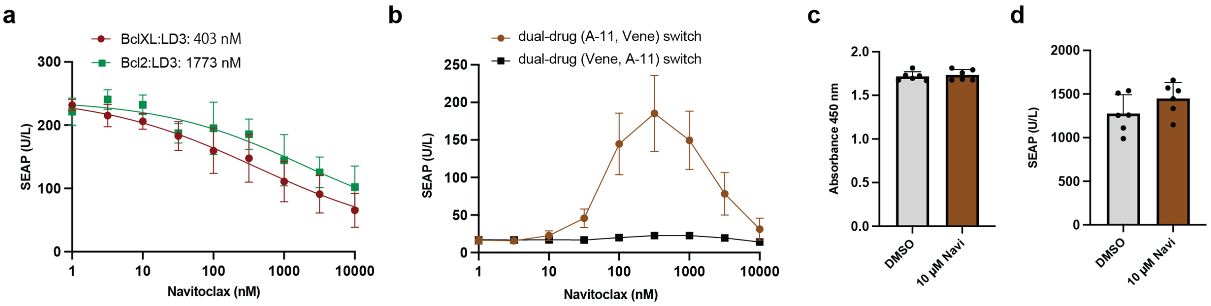

Supplementary Figure 3: Navitoclax used to construct CBP$_{Navi}$.

a) Dose responses of cells expressing GEMS receptors with BclXL:LD3 (PSV40-IgK-BclXL-EpoRm-IL-6RBm-pA and PSV40-IgK-LD3-EpoRm-IL-6RBm-pA) and Bcl2:LD3 (PSV40-IgK-Bcl2-EpoRm-IL-6RBm-pA and PSV40-IgK-LD3-EpoRm-IL-6RBm-pA) complexes disrupting by Navitoclax. **b)** Dose responses to Navitoclax of cells expressing the GEMS with dual-drug (A-1155463, Venetoclax) controlled switch and GEMS with dual-drug (Venetoclax, A-1155463) controlled switch. Each data point represents the mean ± s.d. of three replicates. **c-d)** Navitoclax effects on cell viability and protein production of HEK293T cells. HEK293T cells were transfected with a plasmid for constitutive SEAP production and incubated with indicated drugs for 24 hours. SEAP quantifications were performed to measure the protein production and WST-8 was used to test the cell proliferation. For cell proliferation assay, WST-8 was added after 24 hours of drug incubation, and measured its absorbance at 450 nm 4 hours later. Each bar represents the mean of six biological replicates ± s.d., overlaid with a scatter dot plot of the original data points.

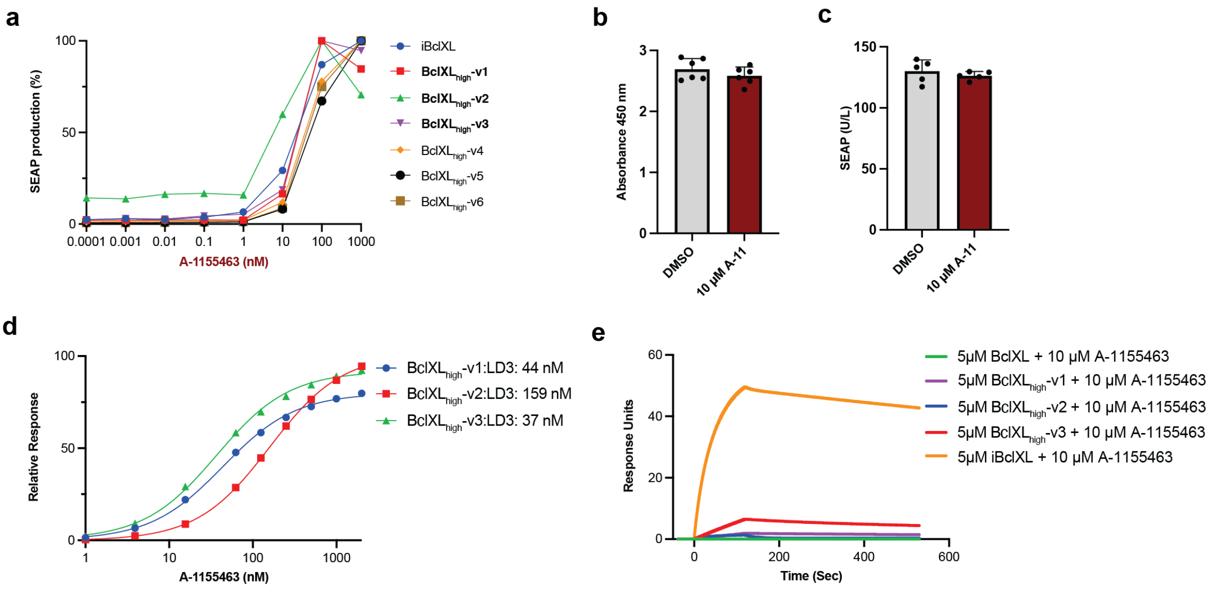

Supplementary Figure 4: Design, screening and validation of BclXL$_{high}$ variants.

a) Screening of BclXL$_{high}$ variants in the GEMS platform. The designed binder LD3 was fused to the drug$_{low}$-receptor (PSV40-IgK-BclXL-LD3-EpoRm-IL-6RBm-pA) in one EpoR chain and BclXL$_{high}$ variants with the insensitive BclXL (iBclXL) as control in the other EpoR chain (PSV40-IgK-BclXL$_{high}$-

EpoRm-IL-6RBm-pA). HEK293T cells were transfected with indicated plasmids, A-1155463 drug ranging from 1 pM to 1 μM were added 12 hours post-transfection, then SEAP was measured 24 hours after drug treatment. **b-c)** A-1155463 effects on cell viability and protein production of HEK293T cells. HEK293T cells were transfected with a plasmid for constitutive SEAP production and incubated with indicated drugs for 24 hours. SEAP quantification were performed to measure the protein production and WST-8 was used to test the cell proliferation. For cell proliferation assay, WST-8 was added after 24 hours of drug incubation, and measured its absorbance at 450 nm 4 hours later. Each bar represents the mean of six biological replicates ± s.d., overlaid with a scatter dot plot of the original data points. **d)** Dissociation constant measurement of $BclXL_{high}$-v(1-3) and LD3. Equilibrium curves are fitted using three-parameter non-linear regression. **e)** Comparison of A-1155463 sensitivity of $BclXL_{high}$-v(1-3) measured by SPR. 5 μM of BclXL or indicated variants were mixed with 10 μM of A-1155463 and injected over the LD3 immobilized chip to analyse the binding response. iBclXL remained stably bound to LD3 (orange), and BclXL was completely inhibited (green). $BclXL_{high}$-v1 (purple), $BclXL_{high}$-v2 (blue), $BclXL_{high}$-v3 (red) showed intermediate binding responses in the presence of 10 μM A-1155463.

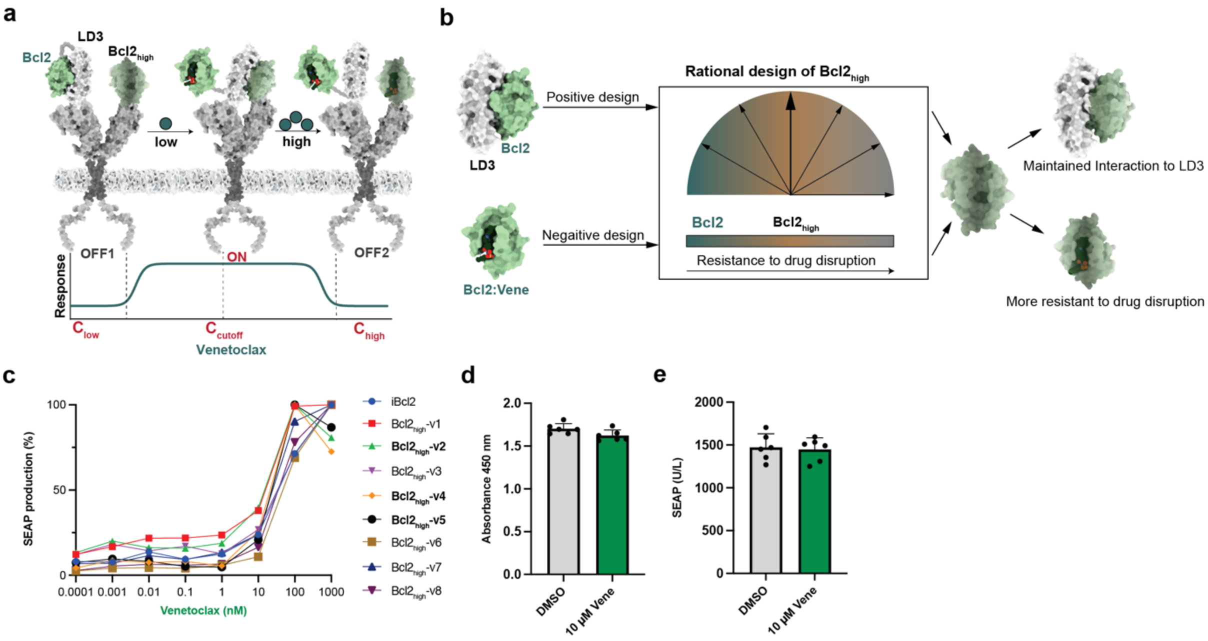

Supplementary Figure 5: Design, screening and validation of Venetoclax controlled bandpass filters.

a) $Bcl2_{high}$-based bandpass filter designs were developed and to respond to different concentrations of Venetoclax. **b)** Multistate design for tuning drug resistance of drug-receptor protein towards to its corresponding inhibitor. **c)** Screening of $Bcl2_{high}$-based bandpass filter variants in the GEMS platform. The designed binder LD3 was fused to the $drug_{low}$-receptor (PSV40-IgK-Bcl2-LD3-EpoRm-IL-6RBm-pA) in one EpoR chain and $Bcl2_{high}$ variants with the insensitive Bcl2 (iBcl2) as control in the other EpoR chain (PSV40-IgK-$Bcl2_{high}$-EpoRm-IL-6RBm-pA). HEK293T cells were transfected with indicated plasmids, A-1155463 drug ranging from 1pM to 1 μM were added 12 hours post-transfection, then SEAP was measured 24 hours after drug treatment. **d-e)** Venetoclax effects on HEK293T cells. HEK293T cells were transfected with a plasmid for constitutive SEAP production and incubated with indicated drugs for 24 hours. SEAP quantification were performed to measure the protein production and WST-8 was used to test the cell proliferation. For cell proliferation assay, WST-8 was added after 24 hours of drug incubation, and measured its absorbance at 450 nm 4 hours later. Each bar represents the mean of six biological replicates ± s.d., overlaid with a scatter dot plot of the original data points.

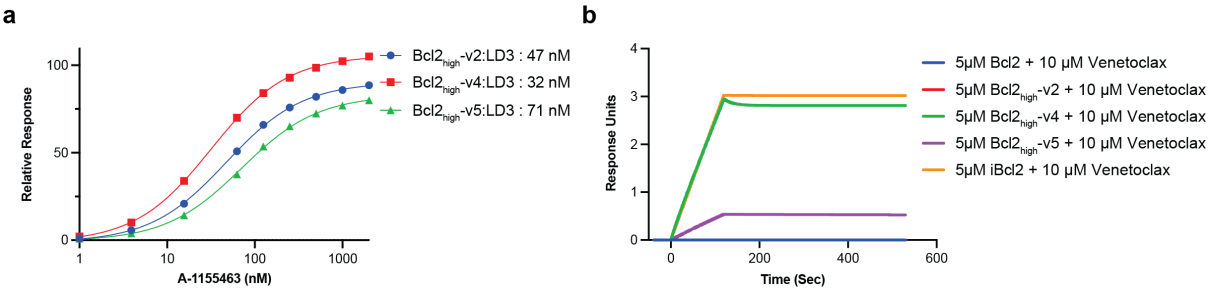

Supplementary Figure 6: Binding affinities and drug resistances of Bcl2$_{high}$-v(2,4,5) assessed by SPR.

a) Measurement of dissociation constants for Bcl2$_{high}$-v(2,4,5) and LD3 interactions. Equilibrium curves are fitted using three-parameter non-linear regression. **b)** Comparison of A-1155463 resistance of Bcl2$_{high}$-v(2,4,5) measured by SPR. 5 μM of Bcl2 or indicated variants were mixed with 10 μM of Venetoclax and injected over an LD3 immobilized chip to analyse the binding response. iBcl2 remained the highest response (orange), and Bcl2 was completely inhibited (blue). Bcl2$_{high}$-v2 (red), Bcl2$_{high}$-v4 (green), Bcl2$_{high}$-v5 (purple) showed intermediate binding responses in the presence of 10 μM Venetoclax.

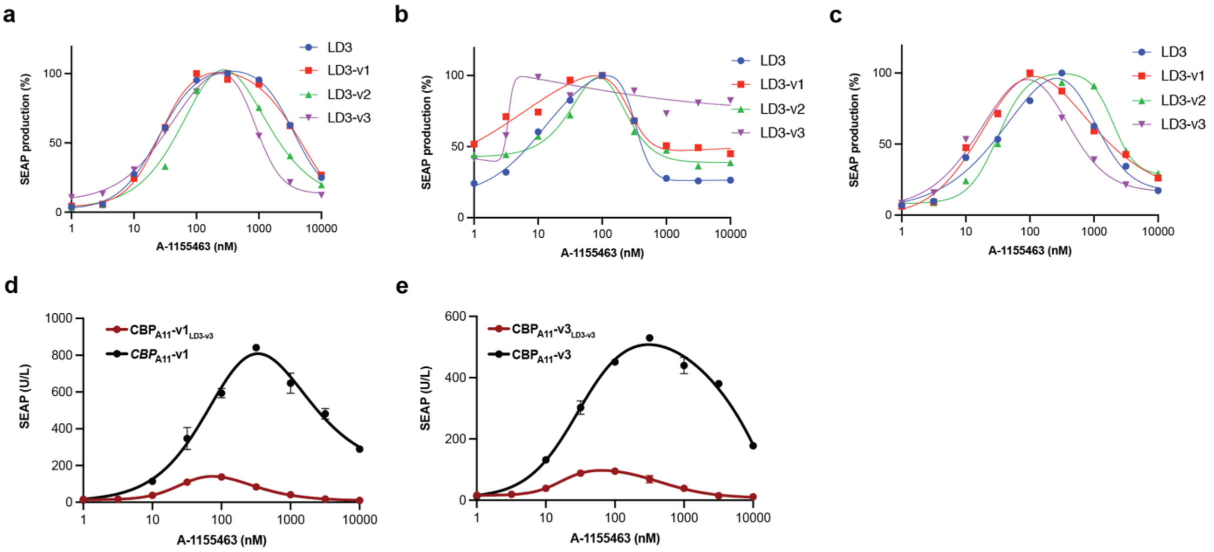

Supplementary Figure 7: Screening of LD3 variants in CBP$_{A11}$-v(1-3) and raw data of CBP$_{A11}$-v(1,3) with LD3-v3 variant.

a-c) Screening of LD3 and LD3-v(1-3) variants in complex with BclXL for more sensitive CBP$_{A11}$-v1 (a), CBP$_{A11}$-v2 (b), CBP$_{A11}$-v3 (c) in the GEMS platform. The LD3 variants replaced the role of LD3 (PSV40-IgK-BclXL-LD3-v(1-3)-EpoRm-IL-6RBm-pA) in one EpoR chain and BclXL$_{high}$-v(1-3) in the other EpoR chain (PSV40-IgK-BclXL$_{high}$-V(1-3)-EpoRm-IL-6RBm-pA). HEK293T cells were transfected with indicated plasmids, A-1155463 drug ranging from 1 pM to 1 μM were added 12 hours post-transfection, then SEAP was measured 24 hours after drug treatment. **d)** Dose responses of CBP$_{A11}$-v1 compared with CBP$_{A11}$-v1$_{LD3-v3}$ in engineered cells. Each data point represents the mean ±s.d. of three replicates and the curves were calculated using Bell-shaped fitting. **e)** Dose responses of CBP$_{A11}$-v3 compared

with CBP$_{A11}$-v3$_{LD3-v3}$ in engineered cells. Each data point represents the mean ±s.d. of three replicates and the curves were calculated using Bell-shaped fitting.

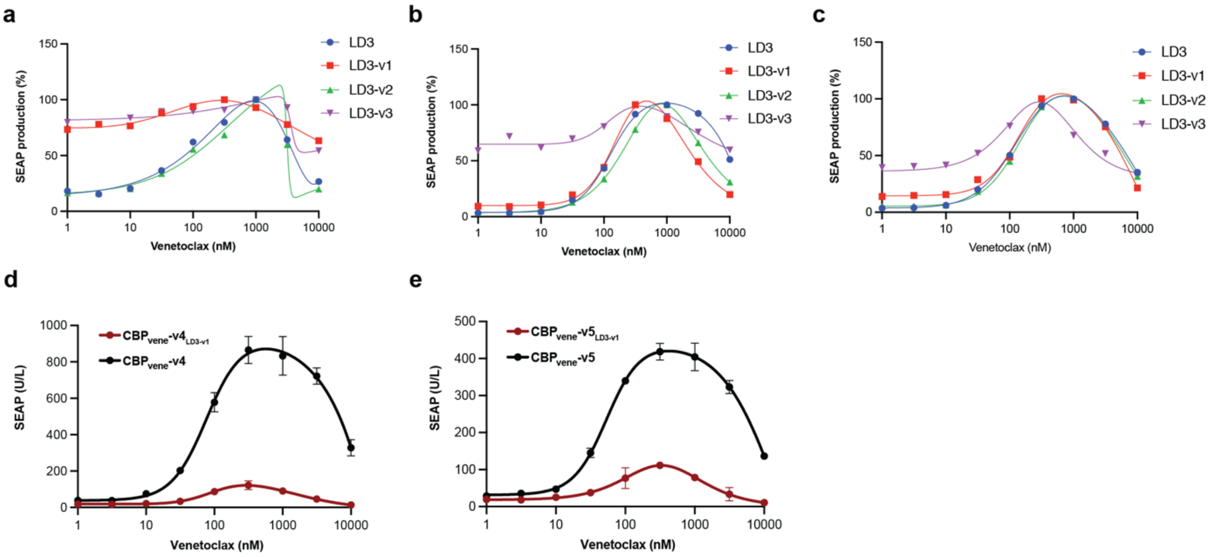

Supplementary Figure 8: Screening of LD3 variants in CBP$_{vene}$-v(2,4,5) and raw data of CBP$_{vene}$-v(4,5) with LD3-v1.

a-c) Screening of LD3 and LD3-v(1-3) variants in complex with Bcl2 for more sensitive CBP$_{vene}$-v2 (a), CBP$_{vene}$-v4 (b), CBP$_{vene}$-v5 (c) in the GEMS platform. The LD3 variants replaced the role of LD3 (PSV40-IgK-Bcl2-LD3-v(1-3)-EpoRm-IL-6RBm-pA) in one EpoR chain and Bcl2$_{high}$-v(2,4,5) in the other EpoR chain (PSV40-IgK-Bcl2$_{high}$-v(2,4,5)-EpoRm-IL-6RBm-pA). HEK293T cells were transfected with indicated plasmids, A-1155463 drug ranging from 1 pM to 1 μM were added 12 hours post-transfection, then SEAP was measured 24 hours after drug treatment. **d)** Dose responses of CBP$_{vene}$-v4 compared with CBP$_{vene}$-v4$_{LD3-v1}$ in engineered cells. Each data point represents the mean ± s.d. of three replicates and the curves were calculated using Bell-shaped fitting. **e)** Dose responses of CBP$_{vene}$-v5 compared with CBP$_{vene}$-v5$_{LD3-v1}$ in engineered cells. Each data point represents the mean ± s.d. of three replicates and the curves were calculated using Bell-shaped fitting.

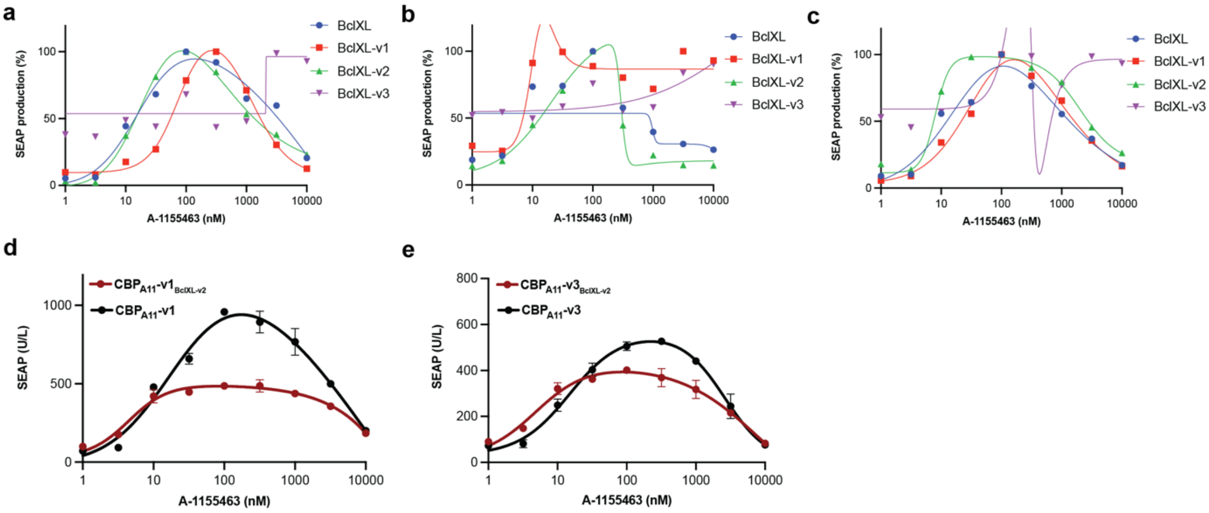

Supplementary Figure 9: Screening of BclXL Alamut variants in CBP$_{A11}$-v(1-3) and raw data of CBP$_{A11}$-v(1,3) with BclXL-v2.

a-c) Screening of BclXL and BclXL-v(1-3) variants in complex with LD3 for better CBP$_{A11}$-v1 (a), CBP$_{A11}$-v2 (b), CBP$_{A11}$-v3 (c) in the GEMS platform. The BclXL variants replaced the role of LD3 (PSV40-IgK-BclXL-v(1-3)-LD3-EpoRm-IL-6RBm-pA) in one EpoR chain and BclXL$_{high}$-v(1-3) in the other EpoR chain (PSV40-IgK-BclXL$_{high}$-v(1-3)-EpoRm-IL-6RBm-pA). HEK293T cells were transfected with indicated plasmids, A-1155463 drug ranging from 1 pM to 1 μM were added 12 hours post-transfection, then SEAP was measured 24 hours after drug treatment. **d)** Drug dose-dependent responses of CBP$_{A11}$-v1 compared with CBP$_{A11}$-v1$_{BclXL\text{-}v2}$ in engineered cells. Each data point represents the mean ±s.d. of three replicates and the curves were calculated using Bell-shaped fitting. **e)** Drug dose-dependent responses of CBP$_{A11}$-v3 compared with CBP$_{A11}$-v3$_{BclXL\text{-}v2}$ in engineered cells. Each data point represents the mean ±s.d. of three replicates and the curves were calculated using Bell-shaped fitting.

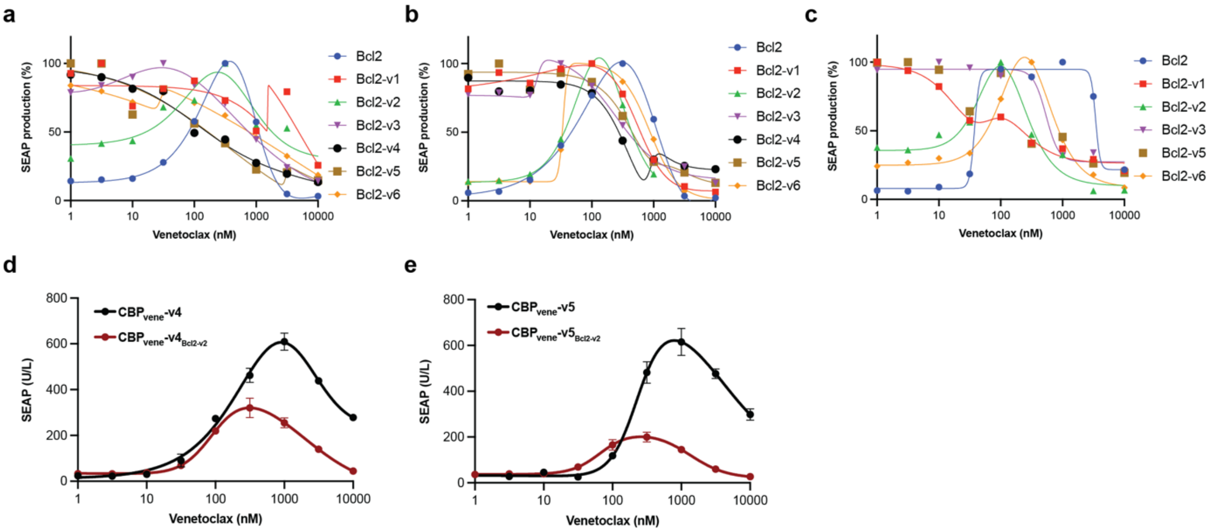

Supplementary Figure 10: Screening of Bcl2 variants in CBP$_{vene}$-v(2,4,5) and raw data of CBP$_{vene}$-v(2,4,5) with Bcl2-v2.

a-c) Screening of Bcl2 and Bcl2-v(1-6) variants in complex with Bcl2 for more sensitive CBP$_{vene}$-v2 (a), CBP$_{vene}$-v4 (b), CBP$_{vene}$-v5 (c) in the GEMS platform The LD3 variants replaced the role of LD3 (PSV40-IgK-Bcl2-v(1-6)-LD3-EpoRm-IL-6RBm-pA) in one EpoR chain and Bcl2$_{high}$-v(2,4,5) in the other EpoR chain (PSV40-IgK-Bcl2$_{high}$-v(2,4,5)-EpoRm-IL-6RBm-pA). HEK293T cells were transfected with indicated plasmids, A-1155463 drug ranging from 1pM to 1 μM were added 12 hours post-transfection, then SEAP was measured 24 hours after drug treatment. **d)** Drug dose-dependent responses of CBP$_{vene}$-v4 compared with CBP$_{vene}$-v4$_{Bcl2\text{-}v2}$ in engineered cells. Each data point represents the mean ±s.d. of three replicates and the curves were calculated using Bell-shaped fitting. **e)** Drug dose-dependent responses of CBP$_{vene}$-v5 compared with CBP$_{vene}$-v5$_{Bcl2\text{-}v2}$ in engineered cells. Each data point represents the mean±s.d. of three replicates and the curves were calculated using Bell-shaped fitting.

Supplementary Table

Supplementary Table 1: Single drug reversible design of BclXL.

Name	Sequences
BclXL$_{high}$-v1	MSQSNRELVVDFLSYKLSQKGYSWSQFSDVEENRTEAPEGTESEAVKQALREAGDEFELRYRRAFSDL**L**SQLHITPGTAYQSFEQVVNELFRDGVNWGRIVAFFSFGG**L**LCVESVDKEMQVLVSRIAAWMATYLNDHLEPWIQENGGWDTFVELYGNNAAAESRKGQER
BclXL$_{high}$-v2	MSQSNRELVVDFLSYKLSQKGYSWSQFSDVEENRTEAPEGTESEAVKQALREAGDEFELRYRRAFSDLTSQLHITPGTAYQSFEQVVNELFRDGVNWGRIVAFFSFGG**V**LCVESVDKEMQVLVSRIAAWMATYLNDHLEPWIQENGGWDTFVELYGNNAAAESRKGQER
BclXL$_{high}$-v3	MSQSNRELVVDFLSYKLSQKGYSWSQFSDVEENRTEAPEGTESEAVKQALREAGDEFELRY**F**RAFSDL**V**SQLHITPGTAYQSFEQVVNELFRDGVNWGRIVAFFSFGGALCVESVDKEMQVLVSRIAAWMATYLNDHLEPWIQENGGWDTFVELYGNNAAAESRKGQER
BclXL$_{high}$-v4	MSQSNRELVVDFLSYKLSQKGYSWSQFSDVEENRTEAPEGTESEAVKQALREAGDEF**S**LRY**E**RA**I**SDLTSQLHITPGTAYQSFEQVVNELFRDGVNWGRIVAFFSFGGALCVESVDKEMQVLVSRIAAWMATYLNDHLEPWIQENGGWDTFVELYGNNAAAESRKGQER
BclXL$_{high}$-v5	MSQSNRELVVDFLSYKLSQKGYSWSQFSDVEENRTEAPEGTESEAVKQALREAGDEFELRY**E**RA**I**SDL**V**SQLHITPGTAYQSFEQVVNELFRDGVNWGRIVAFFSFGGALCVESVDKEMQVLVSRIAAWMATYLNDHLEPWIQENGGWDTFVELYGNNAAAESRKGQER
BclXL$_{high}$-v6	MSQSNRELVVDFLSYKLSQKGYSWSQFSDVEENRTEAPEGTESEAVKQALREAGDEF**S**LRY**E**RA**I**SDL**V**SQLHITPGTAYQSFEQVVNELFRDGVNWGRIVAFFSFGGALCVESVDKEMQVLVSRIAAWMATYLNDHLEPWIQENGGWDTFVELYGNNAAAESRKGQER

Supplementary Table 2: Single drug reversible Bcl2.

Name	Sequences
Bcl2$_{high}$-v1	MAHPGRTGYDNREIVMKYIHYKLSQRGYEWDAGDDVEENRTEAPEGTESEVVHLTLRQAGDDFSRRYRRDFAEMSSQLHLTPFTARGRFATVVEELFRDGVNWGRIVAFFEFGGVMC**I**ESVNREMSPLVDNIALWMTEYLNRHLHTWIQDNGGWDAFVEL**H**GPSMR
Bcl2$_{high}$-v2	MAHPGRTGYDNREIVMKYIHYKLSQRGYEWDAGDDVEENRTEAPEGTESEVVHLTLRQAGD**N**FSRRYRRDFAEMSSQLHLTPFTARGRFATVVEELFRDGVNWGRIVAFFEFGGVMCVESVNREMSPLVDNIALWMTEYLNRHLHTWIQDNGGWDAFVEL**H**GPSMR
Bcl2$_{high}$-v3	MAHPGRTGYDNREIVMKYIHYKLSQRGYEWDAGDDVEENRTEAPEGTESEVVHLTLRQ**T**GD**S**FSRRYRRDFAEMSSQLHLTPFTARGRFATVVEELFRDGVNWGRIVAFFEFGGVMCVESVNREMSPLVDNIALWMTEYLNRHLHTWIQDNGGWDAFVELYGPSMR
Bcl2$_{high}$-v4	MAHPGRTGYDNREIVMKYIHYKLSQRGYEWDAGDDVEENRTEAPEGTESEVVHLTLRQA**V**DDFSRRYRRDFAEMSSQLHLTPFTARGRFATVVEELFRDGVNWGRIVAFFEFGGVMCVESVNREMSPLVDNIALWMTEYLNRHLHTWIQDNGGWDAFVELYGPSMR
Bcl2$_{high}$-v5	MAHPGRTGYDNREIVMKYIHYKLSQRGYEWDAGDDVEENRTEAPEGTESEVVHLTLRQAGD**Y**FSRRYRRDFAEMSSQLHLTPFTARGRFATVVEELFRDGVNWGRIVAFFEFGGVMCVESVNREMSPLVDNIALWMTEYLNRHLHTWIQDNGGWDAFVELYGPSMR

Bcl2$_{high}$-v6	MAHPGRTGYDNREIVMKYIHYKLSQRGYEWDAGDDVEENRTEAPEGTESEVVHLTLR<br>QA**V**D**Y**FSRRYRRDFAEMSSQLHLTPFTARGRFATVVEELFRDGVNWGRIVAFFEFGG<br>VMCVESVNREMSPLVDNIALWMTEYLNRHLHTWIQDNGGWDAFVELYGPSMR
Bcl2$_{high}$-v7	MAHPGRTGYDNREIVMKYIHYKLSQRGYEWDAGDDVEENRTEAPEGTESEVVHLTLR<br>QAGD**E**FSRRYRRDFAEMSSQLHLTPFTARGRFATVVEELFRDGVNWGRIVAFFEFGG<br>VMCVESVNREMSPLVDNIALWMTEYLNRHLHTWIQDNGGWDAFVELYGPSMR
Bcl2$_{high}$-v8	MAHPGRTGYDNREIVMKYIHYKLSQRGYEWDAGDDVEENRTEAPEGTESEVVHLTLR<br>QA**V**D**E**FSRRYRRDFAEMSSQLHLTPFTARGRFATVVEELFRDGVNWGRIVAFFEFGG<br>VMCVESVNREMSPLVDNIALWMTEYLNRHLHTWIQDNGGWDAFVELYGPSMR

Supplementary Table 3: LD3 variants.

Name	Sequences
LD3-v1	QRWELALGRFL**A**YLSWVSTLSEQVQEELLSSQVTQELRALMDETMKELKAYKSELEEQL<br>TPVAEETRARLSKELQAAQARLGADMEDVRGRLVQYRGEVQAMLGQSTEELRVRLASH<br>LIALALRLIGDAFDLQKRLAVY
LD3-v2	QRWELALGRFLEYLSWVSTLSEQVQEELLSSQVTQELRALMDETMKELKAYKSELEEQL<br>TPVAEETRARLSKELQAAQARLGADMEDVRGRLVQYRGEVQAMLGQSTEELRVRLASH<br>LIALAL**A**LIGDAFDLQKRLAVY
LD3-v3	QRWELALGRFLEYLSWVSTLSEQVQEELLSSQVTQELRALMDETMKELKAYKSELEEQL<br>TPVAEETRARLSKELQAAQARLGADMEDVRGRLVQYRGEVQAMLGQSTEELRVRLASH<br>LIALALRLIG**A**AFDLQKRLAVY

Supplementary Table 4: BclXL variants.

Name	Sequences
BclXL-v1	MSQSNRELVVDFLSYKLSQKGYSWSQFSDVEENRTEAPEGTESEAVKQALREAGDEF<br>ELRYRRAFSDLTS**A**LHITPGTAYQSFEQVVNELFRDGVNWGRIVAFFSFGGALCVESV<br>DKEMQVLVSRIAAWMATYLNDHLEPWIQENGGWDTFVELYGNNAAAESRKGQER
BclXL-v2	MSQSNRELVVDFLSYKLSQKGYSWSQFSDVEENRTEAPEGTESEAVKQALREAGDEF<br>ELRYRRAFSDLTSQLHITPGTAYQSFEQVVNE**A**FRDGVNWGRIVAFFSFGGALCVESV<br>DKEMQVLVSRIAAWMATYLNDHLEPWIQENGGWDTFVELYGNNAAAESRKGQER
BclXL-v3	MSQSNRELVVDFLSYKLSQKGYSWSQFSDVEENRTEAPEGTESEAVKQALREAGDEF<br>ELRYRRAFSDLTSQLHITPGTAYQSFEQVVNELFRDGVNWG**A**IVAFFSFGGALCVESV<br>DKEMQVLVSRIAAWMATYLNDHLEPWIQENGGWDTFVELYGNNAAAESRKGQER

Supplementary Table 5: Bcl2 variants.

Name	Sequences
Bcl2-v1	MAHPGRTGYDNREIVMKYIHYKLSQRGYEWDAGDDVEENRTEAPEGTESEVVHLTLR<br>QAGDD**A**SRRYRRDFAEMSSQLHLTPFTARGRFATVVEELFRDGVNWGRIVAFFEFGG<br>VMCVESVNREMSPLVDNIALWMTEYLNRHLHTWIQDNGGWDAFVELYGPSMR
Bcl2-v2	MAHPGRTGYDNREIVMKYIHYKLSQRGYEWDAGDDVEENRTEAPEGTESEVVHLTLR<br>QAGDDFSRRYRRDFAEMSSQLHLTPFTARGRFAT**A**VEELFRDGVNWGRIVAFFEFGG<br>VMCVESVNREMSPLVDNIALWMTEYLNRHLHTWIQDNGGWDAFVELYGPSMR
Bcl2-v3	MAHPGRTGYDNREIVMKYIHYKLSQRGYEWDAGDDVEENRTEAPEGTESEVVHLTLR<br>QAGDDFSRRYRRDFAEMSSQLHLTPFTARGRFATVVE**A**LFRDGVNWGRIVAFFEFGG<br>VMCVESVNREMSPLVDNIALWMTEYLNRHLHTWIQDNGGWDAFVELYGPSMR
Bcl2-v4	MAHPGRTGYDNREIVMKYIHYKLSQRGYEWDAGDDVEENRTEAPEGTESEVVHLTLR<br>QAGDDFSRRYRRDFAEMSSQLHLTPFTARGRFATVVEE**A**FRDGVNWGRIVAFFEFGG<br>VMCVESVNREMSPLVDNIALWMTEYLNRHLHTWIQDNGGWDAFVELYGPSMR
Bcl2-v5	MAHPGRTGYDNREIVMKYIHYKLSQRGYEWDAGDDVEENRTEAPEGTESEVVHLTLR<br>QAGDDFSRRYRRDFAEMSSQLHLTPFTARGRFATVVEELFRDGVNWG**A**IVAFFEFGG<br>VMCVESVNREMSPLVDNIALWMTEYLNRHLHTWIQDNGGWDAFVELYGPSMR
Bcl2-v6	MAHPGRTGYDNREIVMKYIHYKLSQRGYEWDAGDDVEENRTEAPEGTESEVVHLTLR<br>QAGDDFSRRYRRDFAEMSSQLHLTPFTARGRFATVVEELFRDGVNWGRIVAFF**A**FGG<br>VMCVESVNREMSPLVDNIALWMTEYLNRHLHTWIQDNGGWDAFVELYGPSMR
