# Supplementary material for: Protein-based bandpass filters for controlling cellular signaling with chemical inputs
Source: Nat Chem Biol. 2023 Nov 13;20(5):586–93. doi: 10.1038/s41589-023-01463-7 (PMC11062894; doi:10.1038/s41589-023-01463-7)
Supplement: Supplementary file 2 — Reporting Summary [file 41589_2023_1463_MOESM2_ESM.pdf]

nature portfolio

Corresponding author(s): Bruno Emanuel Correia

Last updated by author(s): 16-09-2022

Reporting Summary

Nature Portfolio wishes to improve the reproducibility of the work that we publish. This form provides structure for consistency and transparency in reporting. For further information on Nature Portfolio policies, see our Editorial Policies and the Editorial Policy Checklist.

Statistics

For all statistical analyses, confirm that the following items are present in the figure legend, table legend, main text, or Methods section.

n/a	Confirmed	
☐	☒	The exact sample size (*n*) for each experimental group/condition, given as a discrete number and unit of measurement
☐	☒	A statement on whether measurements were taken from distinct samples or whether the same sample was measured repeatedly
☐	☒	The statistical test(s) used AND whether they are one- or two-sided<br>*Only common tests should be described solely by name; describe more complex techniques in the Methods section.*
☒	☐	A description of all covariates tested
☒	☐	A description of any assumptions or corrections, such as tests of normality and adjustment for multiple comparisons
☐	☒	A full description of the statistical parameters including central tendency (e.g. means) or other basic estimates (e.g. regression coefficient) AND variation (e.g. standard deviation) or associated estimates of uncertainty (e.g. confidence intervals)
☒	☐	For null hypothesis testing, the test statistic (e.g. *F*, *t*, *r*) with confidence intervals, effect sizes, degrees of freedom and *P* value noted<br>*Give P values as exact values whenever suitable.*
☒	☐	For Bayesian analysis, information on the choice of priors and Markov chain Monte Carlo settings
☒	☐	For hierarchical and complex designs, identification of the appropriate level for tests and full reporting of outcomes
☒	☐	Estimates of effect sizes (e.g. Cohen's *d*, Pearson's *r*), indicating how they were calculated

Our web collection on statistics for biologists contains articles on many of the points above.

Software and code

Policy information about availability of computer code

Data collection	Pymol and ChimeraX were used for structural visualization<br>Rosetta was used for protein design.<br>Two rosetta script used in this paper were published before (https://doi.org/10.1038/s41467-021-25735-9)
Data analysis	Graphpad Prism(Version 8.3.0)

For manuscripts utilizing custom algorithms or software that are central to the research but not yet described in published literature, software must be made available to editors and reviewers. We strongly encourage code deposition in a community repository (e.g. GitHub). See the Nature Portfolio guidelines for submitting code & software for further information.

Data

Policy information about availability of data

All manuscripts must include a data availability statement. This statement should provide the following information, where applicable:

- Accession codes, unique identifiers, or web links for publicly available datasets
- A description of any restrictions on data availability
- For clinical datasets or third party data, please ensure that the statement adheres to our policy

The data supporting the findings of this study are available within the article and its Supplementary Information. The codes used in this paper is also published in previous paper (https://doi.org/10.1038/s41467-021-25735-9) and available in github (https://github.com/LPDI-EPFL/CDH_AIR).

Human research participants

Policy information about studies involving human research participants and Sex and Gender in Research.

Reporting on sex and gender	*Use the terms sex (biological attribute) and gender (shaped by social and cultural circumstances) carefully in order to avoid confusing both terms. Indicate if findings apply to only one sex or gender; describe whether sex and gender were considered in study design whether sex and/or gender was determined based on self-reporting or assigned and methods used. Provide in the source data disaggregated sex and gender data where this information has been collected, and consent has been obtained for sharing of individual-level data; provide overall numbers in this Reporting Summary. Please state if this information has not been collected. Report sex- and gender-based analyses where performed, justify reasons for lack of sex- and gender-based analysis.*
Population characteristics	*Describe the covariate-relevant population characteristics of the human research participants (e.g. age, genotypic information, past and current diagnosis and treatment categories). If you filled out the behavioural & social sciences study design questions and have nothing to add here, write "See above."*
Recruitment	*Describe how participants were recruited. Outline any potential self-selection bias or other biases that may be present and how these are likely to impact results.*
Ethics oversight	*Identify the organization(s) that approved the study protocol.*

Note that full information on the approval of the study protocol must also be provided in the manuscript.

Field-specific reporting

Please select the one below that is the best fit for your research. If you are not sure, read the appropriate sections before making your selection.

☒ Life sciences ☐ Behavioural & social sciences ☐ Ecological, evolutionary & environmental sciences

For a reference copy of the document with all sections, see nature.com/documents/nr-reporting-summary-flat.pdf

Life sciences study design

All studies must disclose on these points even when the disclosure is negative.

Sample size	No sample size calculation was performed. For the cell culture experiments, n=3 biological replicates were predicted to be sufficient for estimating the spread of data, for calculating the mean and for detecting statistically significant differences between compared groups. Preliminary results and experience in similar cell culture experiments support n=3 biologically independent samples per individual experiment as sufficient to detect meaningful differences in reporter gene expression.
Data exclusions	We did minimal data exclusion of points where the experiment was technically problematic and the measurements of the reporter proteins yielded negative values. Here is a summary of the excluded data figure 5b (three data points excluded of CBP-A11v1 and CBP-A11v3 measurement, 3 out of 30), figure 5f (three data points excluded of CBPvene-v5, 3 out of 30).
Replication	All cellular experiments were performed with three biological replicates. For reported bandpass curves have been repeated in three independent assays, figure3c, figure 4c-h, figure 5b-c, figure 5e-f.
Randomization	No animal or human research participants were involved. For cell culture experiments, no covariates based on sample allocations to experimental groups could be observed and no randomization was performed. All transfection of drug treated and non-treated, dose-dependent drug treatment were performed with cells transfected under same conditions with the same transfection mix and subsequent addition of drugs. The cells for these experiments were cultured in the same plate with the same well-distribution. For most experiments the inner 60 wells of 96 well plates were used to avoid the effects of evaporation.
Blinding	No animal or human research participants were involved. For cell culture experiments investigators were not blinded. Our workflow makes extensive use of multichannel pipetting for conducting several experiments at the same time. Hence it is unlikely that even subconcious bias regarding anticipated results could influence the data. The parallel conduction of several experiments makes it unlikely that the researcher could identify any given transfection mix, hence elaborate blinding procedures were deemed unnecessary.

Reporting for specific materials, systems and methods

We require information from authors about some types of materials, experimental systems and methods used in many studies. Here, indicate whether each material, system or method listed is relevant to your study. If you are not sure if a list item applies to your research, read the appropriate section before selecting a response.

March 2021

Materials & experimental systems

n/a	Involved in the study
☒	☐ Antibodies
☐	☒ Eukaryotic cell lines
☒	☐ Palaeontology and archaeology
☒	☐ Animals and other organisms
☒	☐ Clinical data
☒	☐ Dual use research of concern

Methods

n/a	Involved in the study
☒	☐ ChIP-seq
☒	☐ Flow cytometry
☒	☐ MRI-based neuroimaging

Eukaryotic cell lines

Policy information about cell lines and Sex and Gender in Research

Cell line source(s)	HEK293T cells
Authentication	The cell line was thawed from previously verified cryo stocks and no further authentication was performed.
Mycoplasma contamination	The cell line was thawed from stocks previously shown to be mycoplasma free and not retested for mycoplasma contamination.
Commonly misidentified lines (See ICLAC register)	None
